# Microbial properties explain temporal variation in soil respiration in a grassland subjected to nitrogen addition

**DOI:** 10.1038/srep18496

**Published:** 2015-12-18

**Authors:** Yue Li, Yinghui Liu, Shanmei Wu, Lei Niu, Yuqiang Tian

**Affiliations:** 1 State Key Laboratory of Earth Surface Processes and Resource Ecology, Beijing Normal University, Beijing 100875,China; 2 Academy of Disaster Reduction and Emergency Management, Beijing Normal University, Beijing 100875, China; 3 College of Resources Science and Technology, Beijing Normal University, Beijing 100875, China

## Abstract

The role of soil microbial variables in shaping the temporal variability of soil respiration has been well acknowledged but is poorly understood, particularly under elevated nitrogen (N) deposition conditions. We measured soil respiration along with soil microbial properties during the early, middle, and late growing seasons in temperate grassland plots that had been treated with N additions of 0, 2, 4, 8, 16, or 32 g N m^−2^ yr^−1^ for 10 years. Representing the averages over three observation periods, total (R_s_) and heterotrophic (R_h_) respiration were highest with 4 g N m^−2^ yr^−1^, but autotrophic respiration (R_a_) was highest with 8 to 16 g N m^−2^ yr^−1^. Also, the responses of R_h_ and R_a_ were unsynchronized considering the periods separately. N addition had no significant impact on the temperature sensitivity (*Q*_10_) for R_s_ but inhibited the *Q*_10_ for R_h_. Significant interactions between observation period and N level occurred in soil respiration components, and the temporal variations in soil respiration components were mostly associated with changes in microbial biomass carbon (MBC) and phospholipid fatty acids (PLFAs). Further observation on soil organic carbon and root biomass is needed to reveal the long-term effect of N deposition on soil C sequestration.

Nitrogen (N) limits terrestrial ecosystem productivity^1^. By the 2000s, China’s atmospheric N deposition had increased to 2 g N m^−2^ yr^−1^ 2. The increase in N deposition concentrations greatly influences terrestrial ecosystem carbon (C) sequestration^3^. Reports on the effects of N addition on different components of soil respiration have been inconsistent. Soil autotrophic respiration (R_a_) and heterotrophic respiration (R_h_) have been found to be positively correlated with N addition with increased fine root biomass^4^,^5^, increased litter input^6^, and the priming effect resulting from the input of labile C substrates^7^, while researchers have also reported that soil respiration components are negatively correlated with N addition with reduced belowground C allocation, mycorrhizal hyphae^4^, microbial biomass^8^ and phenol oxidase concentration^9^.

Environmental conditions (soil texture, temperature and moisture) and organic carbon quality have been considered to be the primary factors for soil carbon mineralization models^10^,^11^. However, the biotic factors such as microbial community structure and activity are closely related to the soil carbon cycle; thus the contribution of soil microbes is gaining attention from researchers^12^. Rates of C sequestration in temperate grassland ecosystems range from 0 to >8 Mg C ha^−1^ yr^−1^^13^. When studying the response of soil respiration to N addition in the grasslands of China, although plant variables (e.g., aboveground biomass, vegetation diversity, and root: shoot ratio) have been commonly measured to reveal the mechanism by which N addition affects soil respiration^4^,^14^,^15^, soil microbial variables (e.g., microbial biomass, microbial community composition and the ratio among microbial groups) have been investigated only infrequently. In addition, previous studies have measured the respiration rate only between 08:00 and 11:00 hours during the day, and the experimental N addition gradients were insufficient^16^,^17^; thus, actual or potential changes in soil respiration in response to N addition may have been misjudged.

In the present study, we measured soil respiration components at an experimental site that had been subjected to six levels of N addition for 10 yr. Soil physicochemical, vegetation and microbial variables were analyzed. Our objectives were to determine how the soil respiration components responded to N deposition during the growing season and how the environmental variables explained those responses. We hypothesized that (1) the responses of soil respiration components to N addition were nonlinear, (2) N addition increased the temperature sensitivity of soil respiration components, and (3) the microbial variables played a substantial role in explaining the response of soil respiration components to the N addition.

## Results

### Respiration components

**Figure 1:**
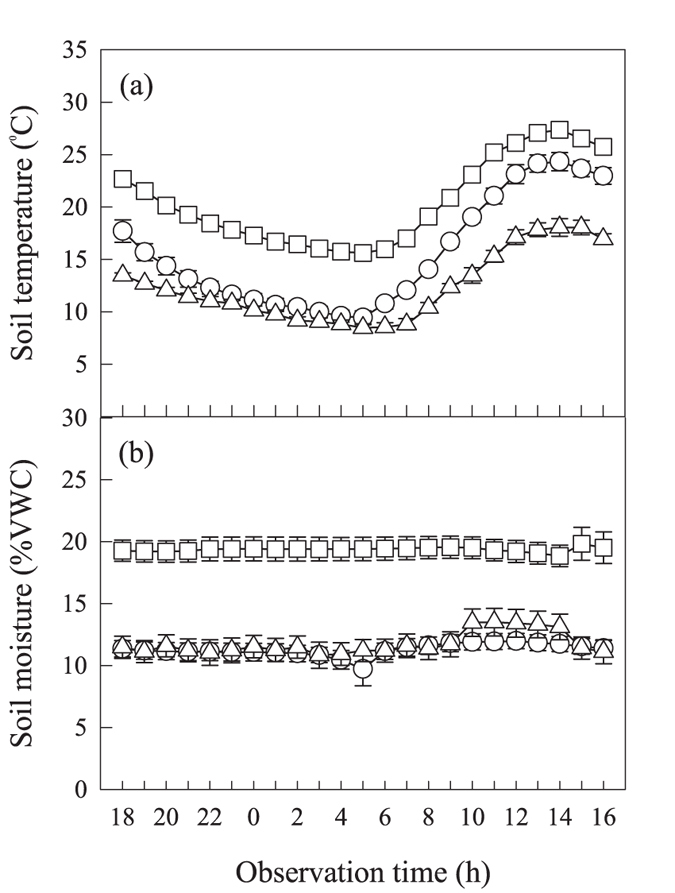
Daily dynamics of soil temperature (**a**) and moisture (**b**) averaged over N levels in the growing season. Circles, squares, and triangles represent soil temperature or moisture in the early, middle and late growing season, respectively. Error bars represent the standard error (n = 3 plot replicates).

Averaged over all plots, the mean soil temperature was 15.6, 20.5, and 12.4 °C, and the mean soil moisture was 10, 18, and 10% VWC (volumetric water content) in the early, middle, and late observation periods, respectively. Although the mean soil temperature was lower in the early than in the middle observation period, the daily temperature variation was largest in the early observation period (Fig. 1). Daily soil moisture variation was relatively small, with ranges of only 2, 1, and 3% in the early, middle, and late observation period, respectively (Fig. 1). The dynamics of soil respiration at six N addition levels followed similar temporal patterns, showing peak and valley occurrences. In the early and late growing season, respiration peaks and valleys occurred at 14:00–16:00 and 5:00–7:00; while in the middle growing season, respiration peaks and valleys occurred at 13:00–15:00 and 3:00–5:00.

**Figure 2:**
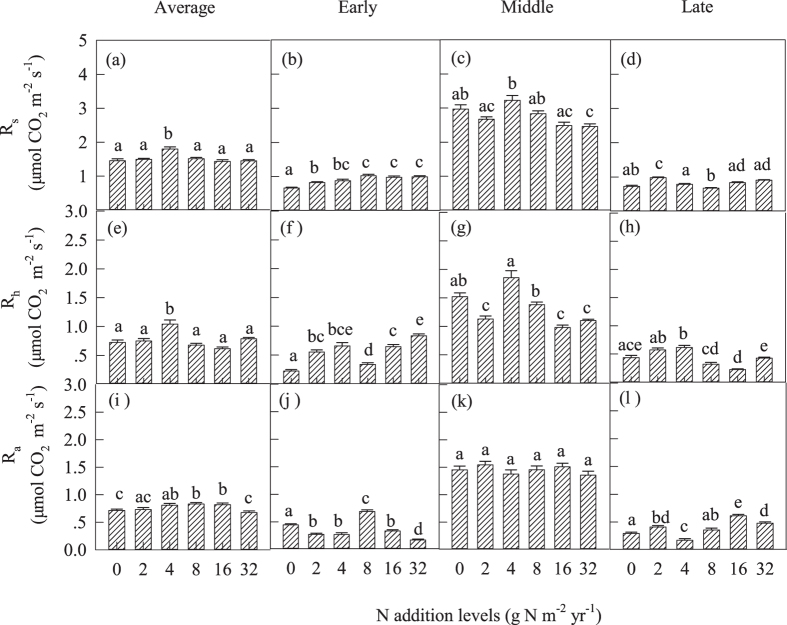
Effects of N addition on soil respiration (**a–d**), heterotrophic respiration (**e–h**) and autotrophic respiration (i–l) averaged across the entire growing season and in the early, middle and late growing season, respectively. R_s_ is soil respiration, R_h_ is heterotrophic respiration, R_a_ is autotrophic respiration. Different letters indicate significant (P < 0.05) differences among N addition levels for each subplot, respectively. Errors bars represent standard error (n = 3 plot replicates).

Respiration components significantly differed among the three observation periods (Table 1). The rates of total soil respiration (R_s_), R_h_ and R_a_ were highest in the middle observation period and were lowest in the late observation period (Fig. 2). Averaged over all plots, R_a_ explained 40, 52, and 46% of R_s_ in the early, middle, and late observation periods, respectively. The effects of N addition on respiration were nonlinear and differed among the respiration components (Table 1). R_s_ and R_h_ were significantly higher, with 4 g N m^−2^ yr^−1^, than with the other N levels, while R_a_ was highest, with 8 to 16 g N m^−2^ yr^−1^ (Fig. 2a,e,i). In the early observation period, R_s_ and R_h_ were generally higher with N addition than without N addition (Fig. 2b,f), while R_a_ was generally inhibited by N addition, except at 8 g N m^−2^ yr^−1^ (Fig. 2j). In the middle observation period, R_s_ insignificantly respond to N addition, except at 32 g N m^−2^ yr^−1^ (Fig. 2c); R_h_ was significantly lower with 2, 16, and 32 g N m^−2^ yr^−1^ than with 0 g N m^−2^ yr^−1^ (Fig. 2g), while R_a_ had no significant response to N addition (Fig. 2k). In the late observation period, R_s_ was slightly but significantly higher with 2 g N m^−2^ yr^−1^ than with the other five levels (Fig. 2d); R_h_ showed no consistent response to N addition (Fig. 2h), while R_a_ was generally stimulated by high levels of N addition (Fig. 2l).

### Sensitivity of respiration to soil temperature and moisture

**Figure 3:**
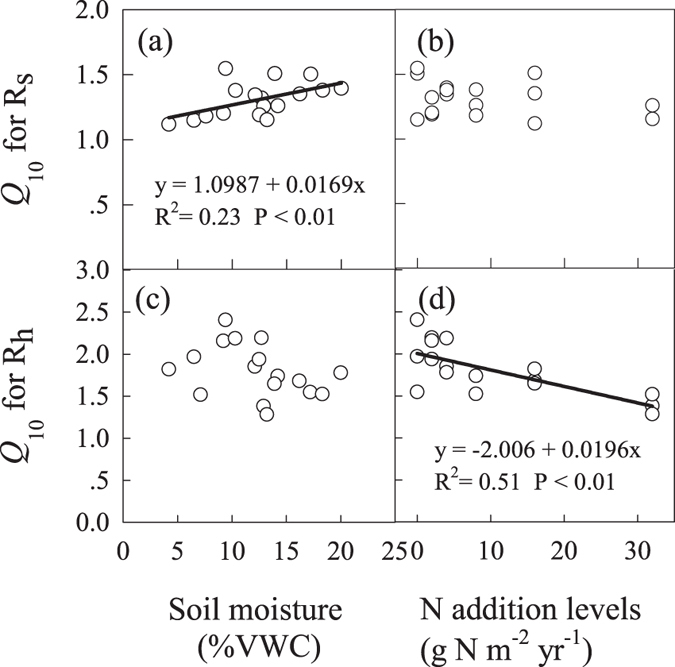
Relationships between the *Q*_10_ temperature coefficient for respiration components and soil moisture (**a,c**) and N addition level (**b,d**) as indicated by linear regression. R_s_ is soil respiration and R_h_ is heterotrophic respiration.

Soil temperature had significantly positive correlations with R_s_ and R_h_ on a daily scale. The *Q*_10_ values for R_s_ ranged between 1.15 and 1.55, and the *Q*_10_ values for R_h_ ranged between 1.28 and 2.41. Because R_a_ was not sensitive to the daily dynamics of the soil temperature in this study, the *Q*_10_ for R_a_ was not calculated. Although the respiration components were not correlated with soil moisture on a daily scale, soil moisture explained 72, 91, and 44% of the variation in R_s_, R_h_, and R_a_ across the entire growing season if all N addition levels were considered. Therefore, in addition to being regulated by soil temperature, respiration was also regulated by soil moisture. The *Q*_10_ for R_s_ increased with soil moisture (Fig. 3a) but was not significantly affected by the level of N addition (Fig. 3b). The *Q*_10_ for R_h_ was unrelated to soil moisture (Fig. 3c) but decreased as N addition increased (Fig. 3d).

### The effects of N addition on soil properties and microorganisms

Comparing the pre-growing and growing season, soil total organic carbon (TOC) and total nitrogen (TN) did not change significantly according to paired-sample *t*-tests. Total phosphorus (TP) was negatively correlated with the N addition level both in the pre-growing and growing season, while TOC, TN, and TOC: TN (C: N) ratio showed no significant pattern in response to the N addition level (Table 2, Supplementary information Table S3). In August, plant aboveground biomass was positively correlated with the N level, but plant species richness was negatively correlated with the N level (Table 2).

Across the entire growing season, soil inorganic nitrogen (NH_4_^+^ -N and NO_3_^−^-N) and dissolved organic carbon (DOC) were positively correlated with the N level, while pH value and microbial biomass carbon (MBC) were negatively correlated with the N level (Table 2). The highest inorganic N content in the middle growing season indicated urea hydrolysis occurred after N addition, but the difference of pH between the early and middle growing season was insignificant (P = 0.167) according to the paired-sample *t*-tests, suggesting the change of pH caused by urea hydrolysis were ignorable (Supplementary information Figure S2 and S3). DOC was highest in the middle growing season, while MBC peaked in the late observation period.

Although the abundance of soil phospholipid fatty acids (PLFAs) and the fungi: bacteria (F: B) ratio were not correlated with the N addition level when data were pooled across the entire growing season, the microbial community composition was changed by N addition in different observation periods. In the middle observation period, negative correlations occurred between microbial community PLFAs and N levels (Table 2). With increases in the N level, the monoenoic: saturated PLFA ratio decreased in the middle observation period, and the F: B ratio decreased in the late observation period (Table 2).

### The relationship between environmental variables and respiration

**Figure 4:**
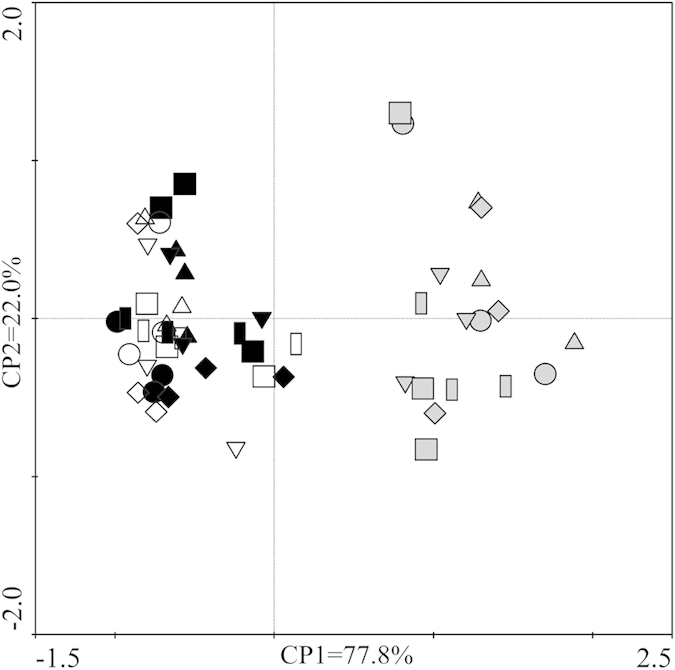
Principal component analysis (PCA) of soil respiration components in the growing season. Circles, upward triangles, boxes, diamonds, downward triangles, and squares represent respiration with 0, 2, 4, 8, 16, and 32 g N m^−2^ yr^−1^ plot, respectively. Black, grey, and white symbols represent respiration in the early, middle and late growing season, respectively.

In a PCA based on 54 samples (18 plots × three observation periods), the first axis explained 77.8% of the variance in the respiration data, whereas the second axis explained 20.0% (Fig. 4). The PCA showed a clear separation of plots over the early, middle and late growing seasons.

**Figure 5:**
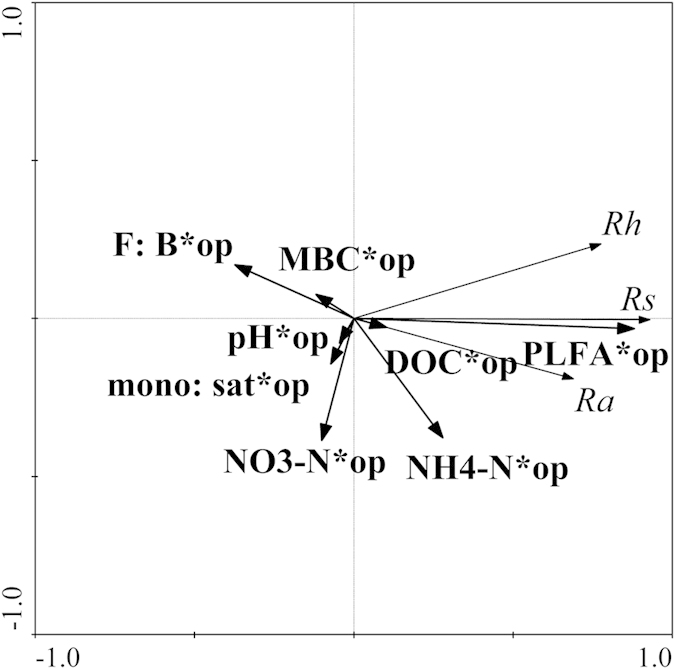
Redundancy analysis (RDA) of soil respiration components in the growing season. MBC is microbial biomass carbon, DOC is dissolved organic carbon, NH_4_^+^-N is ammonium nitrogen, NO_3_^−^-N is nitrate nitrogen, mono: sat is monoenoic: saturated PLFAs ratio, F: B is fungi: bacteria ratio, pH is soil pH value, op is observation period.

Using the interaction between observation period and environmental variables, we examined how the interaction changed relative to the average change in soil respiration components during the growing season. The first two axes of the RDA explained 68.7% of the variance in the relationship between soil respiration components and environmental variables (Fig. 5). Soil respiration components were positively correlated with PLFAs, while respiration was negatively correlated with MBC (Fig. 5).The effect of each environmental variable on the soil respiration components was identified. Among the variables, PLFAs and MBC explained most of the temporal changes in the soil respiration components (Table 3).

## Discussion

Averaged across the entire growing season, the threshold was 4 g N m^−2^ yr^−1^ for R_h_, but 8–16 g N m^−2^ yr^−1^ for R_a_. Considering the three observation periods separately, the responses of R_s_ and R_a_ to N addition were unsynchronized in each period. The increase in R_s_ in the early observation period in response to N addition primarily resulted from an increase in R_h_ rather than R_a_. This result is reasonable because soil microorganisms become active earlier in the growing season than do plants, and accumulation of DOC from the previous year can stimulate R_h_. The correlation between DOC and N level in the early growing season most likely resulted from an increase in litter input or microbial cell lysis^18^,^19^. Inorganic N content was highest after N addition in the middle observation period, a period when R_h_ was substantially inhibited by N addition. Research has demonstrated that N fertilizer reduces the synthesis of various energy-consuming oxidative enzymes, such as phenol oxidase and peroxidase^9^,^20^, leading to a limitation of substrate resources and a decrease in soil respiration. Hence, there may exist one phenomenon of starvation-survival, which is defined as a physiological state resulting from the insufficient energy (catabolism) and nutrients (anabolism) availability for microbial growth and reproduction^21^,^22^. Microbial starvation was induced by N addition in the middle growing season, indicated by the negative correlation between MBC and N level, the failure of DOC to increase with N addition, and the decrease in the ratio of monoenoic: saturated PLFAs with N addition. The promotion of R_a_ by N addition was reported to be caused by increased root biomass^23^. However, previous research at this study site had concluded that root biomass did not increase with N addition^24^. N deposition can switch C_4_ plants to C_3_ plants^25^ and induce the replacement of K-strategy species (perennial grasses and forbs) by r-strategy species (early successional annuals)^26^. Decreases in plant species richness as a result of N addition have been shown in this study. We suspect that no significant response of R_a_ to N addition resulted from the changes in root physiology as the plant community changed^27^. Late in the growing season, R_s_ was insignificantly different among N addition conditions except for 2 g N m^−2^ yr^−1^, due to the balance between R_a_ and R_h_ with 4 to 32 g N m^−2^ yr^−1^. The decrease in the F: B ratio suggests that the N addition dampens the fungi more than bacteria, thus making the microbial community prefer to use the relatively labile soil organic matter^28^,^29^.

The *Q*_10_ value for R_s_ and R_h_ presented different responses to soil moisture and N addition. The *Q*_10_ value for R_s_ at our study site ranged from 1.15 to 1.55, which is consistent with a previous study conducted in Inner Mongolia, China^30^. The positive correlation between soil moisture and the *Q*_10_ value for R_s_ is consistent with previous reports^31^,^32^. However, in contrast to the findings from a previously published incubation experiment conducted at this study site^33^, the *Q*_10_ value for R_h_ showed no clear response to soil moisture, suggesting that N addition may interfere the microbial response of *Q*_10_ to soil moisture. Our original hypothesis is that the N addition promotes the temperature sensitivity of soil respiration because the N limitation could be relieved for plant and microbes^34^,^35^. Surprisingly, N addition showed no clear effect on the *Q*_10_ value for R_s_, and the *Q*_10_ value for R_h_ was significantly reduced by N addition in this study. Mo *et al.*^36^ considered that the differed response of R_s_ *Q*_10_ values to N addition mainly result from the variance in soil nutrient content among the study sites. In addition, Microbial groups have been reported to have different preferences for soil temperature, moisture, and substrate^7^,^37^. The *Q*_10_ value for R_h_ was reported to be higher in soils with high F: B ratios in an incubation experiment^38^. Therefore, we suspect that the low content of TOC in the study site and varying levels of tolerance to N addition among the microbial community might explain the response of the *Q*_10_ value for R_s_ and R_h_ to N addition.

Peaked in the middle growing season, R_a_ had a significantly positive correlation with daily mean soil temperature considering the whole growing season (*R* = 0.876, *P* < 0.001). However, due to its complex structure and adaptation mechanisms involved, plant might be more homeostatic than microbes to short-term environmental change, such as the 24 h dynamics of soil temperature in this study. Besides, the suppression in mycorrhizal respiration may also inhibit the sensitivity of R_a_ to the daily changes in soil temperature, since the arbuscular mycorrhizal fungi (AMF) were found significantly reduced by N addition in the middle observation period, consistent with previous studies^39^.

The N thresholds were found to differ among the microbial variables and plant variables in this study. MBC was low throughout the growing season in plots treated with 8–32 g N m^−2^ yr^−1^, which is consistent with the finding that the critical level of N deposition for MBC is <5 g N m^−2^ yr^−1^ for typical temperate steppes in China^40^. Moreover, nonlinear responses to N addition have also been reported for MBC and plant biomass in other experiments^26^,^29^. In contrast, the 32 g N m^−2^ yr^−1^ did not seem to be a saturation point for the response of aboveground biomass, although plant species richness was inhibited by N addition.

The interactive effects of environmental variables and observation periods were considered on soil respiration components. Time is treated as a potential regulator because it can gradually modify the environmental variables at long term scale. In other words, soil respiration is regulated by the legacy effect of environmental variables subjected to N addition. MBC and PLFAs explained most of the temporal changes in the soil respiration components in the current study. Therefore, we recommend that soil microbial variables (e.g., microbial biomass, microbial community composition and the ratio among microbial groups) be more commonly investigated to reveal the mechanism by which N addition affects soil respiration.

Variation occurs in the responses of belowground carbon compartments to N addition. The correlation between N level and DOC changed through the growing season, while TOC was not correlated with N level, the increase in DOC did not contribute to changes in TOC because DOC is low in the study site and is generally suggested to have minor contribution for C sequestration^41^. TOC stays relatively stable between the pre- and middle growing season, and one study in 2011 concluded that root biomass had no significant response to N addition at this study site^24^. It may be too hasty to draw the conclusion that nitrogen has no significant impact on soil C after 10-year N addition, because we only studied soil nutrient contents for one growing season. In previous studies at the same study site, we also found soil organic matter had no significant difference among N levels after 3-year and 8-year N addition, respectively^24^,^42^. Although the effects of N addition on soil C storage might differ across studies, possibly due to site variation in soil texture^43^, the lack of response of soil C to N addition also occurred in other grassland ecosystems^44^,^45^. It is well acknowledged that N deposition substantially influences terrestrial ecosystem C sequestration, but a point of view is raised that N addition enhances aboveground C rather than soil C^46^. Therefore, information on soil organic carbon and root biomass need to be consistently traced, in order to reveal the effect of N on soil C sequestration at long term scale.

Soil pH value should be taken into consideration in N deposition experiments, although soil pH has no significant impact on the temporal variability of soil respiration components in this study. Previous study has concluded that two potential regulators by N addition should be addressed: N availability and soil acidification^41^, it is possible that soil acidification indirectly impacts soil respiration by shifting the plant community to species with higher acid tolerance and higher specific root respiration^47^,^48^, suppressing soil microbial biomass, enzymatic activity^49^,^50^ and changing the microbial structure to lower F: B ratio^48^,^51^.

Plot trenching is commonly used to estimate R_h_ and R_a_ because of its simplicity and low cost. However, there are inaccuracies in the methodology of trenching that need to be considered. Although we endeavored to minimize the effect, soil disturbance would accelerate the decomposition of soil organic matter which exposed to the air. Soil microbial structure and function will be affected when the plant-derived carbon (e.g., plant residues and rhizodeposition), which stimulates the priming effect of soil organic matter^52^,^53^, changes in the trenched plot. In addition to substrate availability, changes in soil temperature and moisture caused by trenching also regulate soil microbial activity^54^. In fact, roots can both affect R_a_ and R_h_ in the soil, for the rhizosphere-derived CO_2_ results from root respiration, rhizo-microbial respiration, microbial respiration of dead plant residues and additional SOM-derived CO_2_ via the priming effect^55^,^56^. Grass root severing in the trenched plots can result in an inaccuracy in measuring R_h_ because substrate quality is changed by increased dead root decomposition occurring with decreased photosynthates from aboveground^56^. Normally, a lack of photosynthates would be significant only after a long period of stabilization for a trenched plot^56^. Trenching would lead to the overestimation of R_h_ in which the CO_2_ derived from the extra root decomposition was included.

## Methods

### Study area

The study area is located in Duolun County, Inner Mongolia, China (42.02°N, 116.17°E; 1,341 m a.s.l.). The annual mean temperature is 2.1°C, and the mean annual precipitation is 385.5 mm; the typical soil in this region is chestnut, composed of 62.8% sand, 20.3% silt, and 17.0% clay, the bulk density is 1.31 g cm^−3^, and the dominant vegetation species are *Stipa krylovii*, *Leymus chinensis*, *Artemisia frigida, Agropyron cristatum* and *Allium bidentatum*^24^. The growing season in the study site is proximately from May to September. Phenophase of budding mainly occurs from April to June; inflorescence, tasseling and flowering occurs from July to August; And fruiting to senescing occurs from September to October^57^.

The N addition experiment included six N levels (0, 2, 4, 8, 16, and 32 g N m^−2^ yr^−1^) and three replicates for each level. The replicates were assigned to 18 plots (15 × 10 m, separated from each other by a 4-m-wide buffer zone) following a Latin square design (Supplementary Figure S1). In mid-July (during the rainy season) of each year, referred to the weather forecast, dry urea (CO(NH_2_)_2_) has been manually spread on the surface of the plots before the rain since July 2003.

### Respiration measurement

Soil heterotrophic respiration (R_h_, μmol of CO_2_ m^−2^ s^−1^) was measured using the trenching method. On 23 April, 2013, one 40 cm × 40 cm subplot was formed in each plot by inserting iron plates 30 cm deep into the soil. The aboveground vegetation in the subplots was clipped, and the plant tillers and roots in the top 0–10 cm of the soil were carefully removed. In order to maximize the soil integrality in the subplots, instead of sieving roots out of the whole soil block, we only yanked out the clods in the top 0–10 cm of the soil where plant tillers were situated (the magnitude of the clod depended on the tiller). The plant tillers and the roots in the clods were removed before the rootless soil was subsequently refilled, and the remaining roots in the subplots were left to be decomposed. Weeds above the soil in the subplots were clipped if they existed once every 10 days. Twenty-four h before the observation on 22 May, a PVC collar (20 cm diameter, 14 cm height) was inserted in the middle of each subplot to measure R_h_. A second PVC collar was inserted at a randomly selected location outside of the subplot but inside the plot, with aboveground removed and belowground vegetation retained, to measure soil total respiration (R_s_, μmol of CO_2_ m^−2^ s^−1^). All collars were inserted 6–7 cm deep and left in place after they were inserted into the soil.

Respiration was measured during approximately 14-day periods in the early growing period (from 22 May to 4 June), middle growing period (from 23 July to 4 August), and late growing period (from 20 September to 4 October) in 2013, respectively. During each period, 18 plots were each observed once. The respiration rates in three plots with six collars were measured on each date, and the measurement would be postponed for 1–2 days after precipitation to avoid CO_2_ flux pulse. Respiration rate was measured with a soil C-flux automatic measurement system (LI-COR, NE, USA). Meanwhile, soil temperature (°C) and moisture (volumetric water content, %VWC) in the top 0–10 cm layer of the soil near the collar were measured with an auxiliary sensor attached to the LI-COR 8150, and data for each collar were recorded once every 1 h to track the daily dynamics.

One thing to note here is that soil respiration measured between 08:00 and 11:00 was not suitable to represent the mean respiration for 24 h in this study. We treated the mean respiration for 08:00–11:00 as the univariate predictor to fit the mean respiration for 24 h, with an intercept of zero. The R^2^ values indicated that except for the early growing season (y = 1.002×, R^2^ = 0.79), soil respiration measured between 08:00 and 11:00 failed to represent the mean respiration for 24 h (the middle growing season: y = 0.975×, R^2^ = 0.47; the late growing season y = 1.0469 x, R^2^ = 0.46).

### Sampling and analysis

In April, May, July, and September of 2013, we randomly collected three soil samples (0–20 cm depth) in each plot (outside the subplot); these were combined to form one composite sample per plot. Air-dried soil samples were grind and sieved through 0.25 mm before analysis of total organic carbon (TOC, g kg^−1^), total phosphorus (TP, g kg^−1^) and total nitrogen (TN, g kg^−1^). TOC was measured by the potassium dichromate-oxidation method, TP was measured with a spectrometer (SPECTRO, Kleve, Germany), and TN was measured with an elemental analyzer (Perkin-Elmer, MA, USA).

Fresh soil samples were sieved through 2 mm and prepared to measure soil pH, soil inorganic nitrogen (NH_4_^+^-N and NO_3_^−^-N, mg kg^−1^), microbial biomass carbon (MBC, mg kg^−1^), dissolved organic carbon (DOC, mg kg^−1^) and soil phospholipid fatty acids (PLFAs, μmol g^−1^). Soil pH was measured in a 1: 2.5 (soil: water) suspension. Deionized water was added to the soil, and soil solution was extracted from the soil samples after vortex and centrifuge. Ammonium nitrogen (NH_4_^+^-N) and nitrate nitrogen (NO_3_^−^-N) were measured from the solution using ion chromatograph analyzers (DIONEX, CA, USA).

MBC was determined by the chloroform fumigation-extraction method^58^. Soil samples were divided into the fumigated group (chloroform fumigated for 24 h) and the control group. 0.5 M K_2_SO_4_ solution was used to extract soil organic carbon from both groups. The extracts were measured with a TOC analyzer (Elementar, Hanau, Germany). MBC was determined by multiplying the difference of extracted organic carbon between fumigated and control soil by 0.45, the conversion factor. DOC was determined as the extracted organic carbon in the control soil.

PLFAs were measured referring to Bligh & Dyer^59^. The extracts were measured with gas chromatography/mass spectrometry (Agilent Technologies, CA, USA). Based on previous research^28^,^29^,^39^, the groups of microorganisms were divided into bacteria (Gram-positive bacteria and Gram-negative bacteria), fungi, actinomycetes, and arbuscular mycorrhizal fungi (AMF). Decreased ratio of monoenoic to saturated fatty acids is a useful stress signature to provide the profiles of the starving status of natural microbial communities^60^, thus the ratio of monoenoic: saturated PLFAs was measured in this study (Supplementary Table S1).

In early August of 2013, aboveground plant biomass was removed by clipping in a 1 m × 1 m area randomly located area in each plot (outside of the subplot). The clippings were oven dried at 65°C to a constant weight to assess aboveground plant biomass (g m^−2^). Before drying, the clippings were examined to determine species richness.

### Statistical analysis

Equation (1) was used to estimate R_h_ from the plot used to measure R_s_ in the growing season:


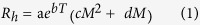


where *R*_*h*_ is the actual heterotrophic respiration rate (μmol CO_2_ m^−2^ s^−1^), *T* is the soil temperature (°C, near the collar used to measure R_h_), and *M* is the soil moisture (%VWC, near the collar used to measure R_h_). Parameters *a* through *d* were estimated, and the resulting equations adequately described the data (Supplementary Table S2). Therefore, the estimated heterotrophic respiration rate is obtained by substituting parameters of *a* through *d*, soil temperature and moisture near the collar (for measuring R_s_) into equation (1). In addition, soil autotrophic respiration (R_a_, μmol CO_2_ m^−2^ s^−1^) is the difference of R_s_ and estimated R_h_.

To evaluate the response of the respiration components to temperature, *Q*_10_ was measured by the following exponential functions:


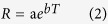



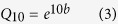


where *R* is either *R*_*s*_, estimated *R*_*h*_ or *R*_*a*_(μmol CO_2_ m^−2^ s^−1^) and *T* is the soil temperature (°C) near the collar used to measure R_s_. Parameter *a* indicates the basal respiration rate, and *b* is the exponent used to calculate *Q*_10_.

A repeated-measures ANOVA was used to assess how the interaction between observation period and the N addition level affected the respiration components. A one-way ANOVA was used to examine the effect of the N addition level on the respiration components, MBC, DOC, inorganic N (NH_4_^+^ and NO_3_^-^), and microbial PLFAs. Paired-sample *t*-tests were used to determine whether the soil properties (TOC, TN, TP and pH) differed between the sampling periods. A Pearson correlation analysis was also used to determine the relationship between N level, inorganic nitrogen, MBC, DOC, PLFAs, and aboveground biomass. The statistical analyses were conducted using IBM SPSS 20.0 (IBM, NY, USA). Graphs were created using Sigmaplot 12.5 (Systat Software, CA, USA).

A principal component analysis (PCA) and a redundancy analysis (RDA) were conducted using CANOCO 4.5 (Microcomputer Power, NY, USA) to determine which aggregates of environmental variables best explained the variances of soil respiration components. Data for three respiration components (R_s_, R_h_, and R_a_) and 54 plots (6 N levels × 3 replicates × 3 observation periods) were used to describe the variances in soil respiration components as affected by N level and observation period, and a split-plot design was used to avoid the autocorrelation between the individual observations in both the respiration and the environmental variables. Monte Carlo tests were used in the automatic forward selection procedure to identify the interactive effects of environmental variable and observation period on soil respiration components.

## Additional Information

**How to cite this article**: Li, Y. *et al.* Microbial properties explain temporal variation in soil respiration in a grassland subjected to nitrogen addition. *Sci. Rep.* **5**, 18496; doi: 10.1038/srep18496 (2015).

## Supplementary Material

Supplementary Information

## Figures and Tables

**Table 1:** Results (*F* values) of repeated-measures analysis of variance for the effects of observation period (op; early, middle, or late), N level (N), and their interaction on the components of respiration for the entire growing season.

Source of variation	Respiration component			
	*df*	R_s_	R_h_	R_a_
op	2	2592.13**	1093.92**	1055.69**
N (g N m^−2^ yr)	5	8.49**	4.68**	4.14**
op × N	10	18.63**	8.08**	17.93**

R_s_ is soil respiration, R_h_ is heterotrophic respiration, R_a_ is autotrophic respiration.

^**^indicates significant difference at *P* < 0.01 (two-tailed).

**Table 2:** Pearson correlation coefficients between environmental variables and N addition level during different observation periods.

Environmental variable	Observation period and N level (g N m^−2^ yr^−1^)					
	April	May	July	August	September	Entire growing season
TOC	0.545	NA	−0.619	NA	NA	NA
TN	0.029	NA	−0.018	NA	NA	NA
TP	−0.946**	NA	−0.757^†^	NA	NA	NA
C: N	0.601	NA	−0.525	NA	NA	NA
pH	NA	−0.906*	−0.910*	NA	−0.957**	−0.940**
Ab	NA	NA	NA	0.936**	NA	NA
Sr	NA	NA	NA	−0.950**	NA	NA
NH_4_^+^-N	NA	0.729^†^	0.871*	NA	0.857*	0.518*
NO_3_^−^-N	NA	0.576	0.895*	NA	0.919**	0.744*
MBC	NA	−0.941**	−0.832**	NA	−0.930**	−0.877**
DOC	NA	0.963**	0.049	NA	0.974**	0.691**
Total FAs	NA	−0.674	−0.825*	NA	−0.468	−0.285
Ba	NA	−0.692	−0.804 ^†^	NA	−0.334	−0.288
G^−^ Ba	NA	−0.656	−0.817*	NA	−0.428	−0.315
G^+^Ba	NA	−0.819*	−0.761 ^†^	NA	−0.402	−0.280
Fu	NA	−0.766 ^†^	−0.822*	NA	−0.654	−0.367
AMF	NA	−0.685	−0.811*	NA	−0.722	−0.376
Ac	NA	−0.493	−0.855*	NA	−0.517	−0.315
F: B	NA	0.421	−0.592	NA	−0.957**	−0.244
Mono:Sat	NA	−0.682	−0.903*	NA	−0.304	−0.662**

TOC is total organic carbon (g kg^−1^), TN is total nitrogen (g kg^−1^), TP is total phosphorus (g kg^−1^), C: N is total organic carbon: total nitrogen ratio, pH is soil pH value, Ab is aboveground biomass (g m^−2^), Sr is Species richness, NH_4_^+^-N is ammonium nitrogen (mg kg^−1^), NO_3_^−^-N is nitrate nitrogen (mg kg^−1^), MBC is microbial biomass carbon (mg kg^−1^), DOC is dissolved organic carbon (mg kg^−1^), total Fas is total PLFAs (μmol g^−1^), Ba is bacteria PLFAs (μmol g^−1^), G^−^ Ba is Gram-negative bacteria PLFAs (μmol g^−1^), G^+^ Ba is Gram-positive bacteria PLFAs (μmol g^−1^), AMF is arbuscular mycorrhizal fungi (μmol g^−1^), Ac is actinomycetes PLFAs (μmol g^−1^), F: B is fungi: bacteria ratio, mono: sat is monoenoic: saturated PLFAs ratio. Significant differences are reported as ^†^*P* < 0.1; *P < 0.05 and **P < 0.01 (two-tailed). NA indicates data not available.

**Table 3:** Marginal and conditional effects of the indicated variables on soil respiration as determined by forward selection in redundancy analysis (RDA).

Variable	Explained α	Explained β	P	F ratio
PLFA*op	0.59	0.59	0.030	68.80
MBC*op	0.01	0.02	0.080	2.44
DOC*op	0.01	0.02	0.136	2.57
NH_4_^+^-N*op	0.09	0.01	0.280	1.77
NO_3_^−^-N*op	0.02	0.02	0.148	2.24
Mono:Sat*op	0.00	0.03	0.594	4.41
F: B*op	0.01	0.00	0.878	0.10
pH*op	0.00	0.00	0.806	0.21

MBC is microbial biomass carbon, DOC is dissolved organic carbon, NH_4_^+^-N is ammonium nitrogen, NO_3_^−^-N is nitrate nitrogen, mono: sat is monoenoic: saturated PLFAs ratio, F: B is fungi: bacteria ratio, pH is soil pH value, op is observation period. Explained α is Marginal effects, which show the variance explained when the variable is used as the only factor. Explained β is Conditional effects, which show the additional variance each variable explains when it is included in the model. P-Level of significance corresponds to β when performing Monte Carlo test.

## References

[b1] Treseder K. K. Nitrogen additions and microbial biomass: a meta-analysis of ecosystem studies. Ecol Lett 11, 1111–1120 (2008). 18673384 10.1111/j.1461-0248.2008.01230.x

[b2] Liu X. J. *et al.* Enhanced nitrogen deposition over China. Nature 494, 459–462 (2013). 23426264 10.1038/nature11917

[b3] Gruber N. & Galloway J. N. An Earth-system perspective of the global nitrogen cycle. Nature 451, 293–296 (2008). 18202647 10.1038/nature06592

[b4] Hasselquist N. J., Metcalfe D. B. & Hogberg P. Contrasting effects of low and high nitrogen additions on soil CO_2_ flux components and ectomycorrhizal fungal sporocarp production in a boreal forest. Global Change Biol 18, 3596–3605 (2012).

[b5] Zhou L. Y. *et al.* Different responses of soil respiration and its components to nitrogen addition among biomes: a meta-analysis. Global Change Biol 20, 2332–2343 (2014). 10.1111/gcb.12490 24323545

[b6] Gentilesca T., Vieno M., Perks M. P., Borghetti M. & Mencuccini M. Effects of Long-Term Nitrogen Addition and Atmospheric Nitrogen Deposition on Carbon Accumulation in Picea sitchensis Plantations. Ecosystems 16, 1310–1324 (2013).

[b7] Bird J. A., Herman D. J. & Firestone M. K. Rhizosphere priming of soil organic matter by bacterial groups in a grassland soil. Soil Biol Biochem 43, 718–725 (2011).

[b8] Jiang X. Y., Cao L. X. & Zhang R. D. Changes of labile and recalcitrant carbon pools under nitrogen addition in a city lawn soil. J Soil Sediment 14, 515–524 (2014).

[b9] Zak D. R., Pregitzer K. S., Burton A. J., Edwards I. P. & Kellner H. Microbial responses to a changing environment: implications for the future functioning of terrestrial ecosystems. Fungal Ecol 4, 386–395 (2011).

[b10] Parton W. J. & Singh J. S. Adapting a biomass simulation-model to a tropical grassland. Ecol Model 23, 151–163 (1984).

[b11] Kemp P. R., Reynolds J. F., Virginia R. A. & Whitford W. G. Decomposition of leaf and root litter of Chihuahuan desert shrubs: effects of three years of summer drought. J Arid Environ 53, 21–39 (2003).

[b12] Garcia-Pausas J. & Paterson E. Microbial community abundance and structure are determinants of soil organic matter mineralisation in the presence of labile carbon. Soil Biol Biochem 43, 1705–1713 (2011).

[b13] Jones M. B. & Donnelly A. Carbon sequestration in temperate grassland ecosystems and the influence of management, climate and elevated CO(2). New Phytol 164, 423–439 (2004).

[b14] Oishi A. C., Palmroth S., Johnsen K. H., Mccarthy H. R. & Oren R. Sustained effects of atmospheric [CO2] and nitrogen availability on forest soil CO2 efflux. Global Change Biol 20, 1146–1160 (2014). 10.1111/gcb.12414 24115580

[b15] Rodriguez A. *et al.* Lability of C in temperate forest soils: Assessing the role of nitrogen addition and tree species composition. Soil Biol Biochem 77, 129–140 (2014).

[b16] Xu W. H. & Wan S. Q. Water- and plant-mediated responses of soil respiration to topography, fire, and nitrogen fertilization in a semiarid grassland in northern China. Soil Biol Biochem 40, 679–687 (2008).

[b17] Yan L. M., Chen S. P., Huang J. H. & Lin G. H. Differential responses of auto- and heterotrophic soil respiration to water and nitrogen addition in a semiarid temperate steppe. Global Change Biol 16, 2345–2357 (2010).

[b18] Breulmann M., Schulz E., Huhn K. W. & Buscot F. O. Impact of the plant community composition on labile soil organic carbon, soil microbial activity and community structure in semi-natural grassland ecosystems of different productivity. Plant Soil 352, 253–265 (2012).

[b19] Henry H. A. L. & Moise E. R. D. Grass litter responses to warming and N addition: temporal variation in the contributions of litter quality and environmental effects to decomposition. Plant Soil 389, 35–43 (2015).

[b20] Cusack D. F., Silver W. L., Torn M. S., Burton S. D. & Firestone M. K. Changes in microbial community characteristics and soil organic matter with nitrogen additions in two tropical forests. Ecology 92, 621–632 (2011). 21608471 10.1890/10-0459.1

[b21] Lever M. A. *et al.* Life under extreme energy limitation: a synthesis of laboratory- and field-based investigations. Fems Microbiol Rev 39, 688–728 (2015). 25994609 10.1093/femsre/fuv020

[b22] Mortia R. Y. The starvation-survival state of microorganisms in nature and its relationship to the bioavailable energy. Experientia 46, 813–817 (1990).

[b23] Zhang C. P. *et al.* Effects of simulated nitrogen deposition on soil respiration components and their temperature sensitivities in a semiarid grassland. Soil Biol Biochem 75, 113–123 (2014).

[b24] Song B., Niu S. L., Li L. H., Zhang L. X. & Yu G. R. Soil carbon fractions in grasslands respond differently to various levels of nitrogen enrichments. Plant Soil 384, 401–412 (2014).

[b25] Zeng D. H. *et al.* Effects of nitrogen addition on vegetation and ecosystem carbon in a semi-arid grassland. Biogeochemistry 98, 185–193 (2010).

[b26] Bai Y. F. *et al.* Tradeoffs and thresholds in the effects of nitrogen addition on biodiversity and ecosystem functioning: evidence from inner Mongolia Grasslands. Global Change Biol 16, 358–372 (2010).

[b27] Laganiere J., Pare D., Bergeron Y. & Chen H. The effect of boreal forest composition on soil respiration is mediated through variations in soil temperature and C quality. Soil Biol Biochem 53, 18–27 (2012).

[b28] Feng X. J. & Simpson M. J. Temperature and substrate controls on microbial phospholipid fatty acid composition during incubation of grassland soils contrasting in organic matter quality. Soil Biol Biochem 41, 804–812 (2009).

[b29] Wei C. Z. *et al.* Nitrogen deposition weakens plant-microbe interactions in grassland ecosystems. Global Change Biol 19, 3688–3697 (2013). 10.1111/gcb.12348 23925948

[b30] Chen Q. S. *et al.* Responses of soil respiration to temperature in eleven communities in Xilingol Grassland, Inner Mongolia. Acta Phytoecologica Sinica 27, 441–447 (2003).

[b31] Rey A., Petsikos C., Jarvis P. G. & Grace J. Effect of temperature and moisture on rates of carbon mineralization in a Mediterranean oak forest soil under controlled and field conditions. Eur J Soil Sci 56, 589–599 (2005).

[b32] Yuste C. J. *et al.* Microbial soil respiration and its dependency on carbon inputs, soil temperature and moisture. Global Change Biol 13, 2018–2035 (2007).

[b33] Li Y., Liu Y. H., Shen W. J., Xu X. & Tian Y. Q. Responses of soil heterotrophic respiration to changes in soil temperature and moisture in a *Stipa krylovii* grassland in Nei Mongol. Acta Phytoecologica Sinica 38, 238–248 (2014).

[b34] Lagomarsino A., De Angelis P., Moscatelli M. C. & Grego S. The influence of temperature and labile C substrates on heterotrophic respiration in response to elevated CO2 and nitrogen fertilization. Plant Soil 317, 223–234 (2009).

[b35] Coucheney E., Stromgren M., Lerch T. Z. & Herrmann A. M. Long-term fertilization of a boreal Norway spruce forest increases the temperature sensitivity of soil organic carbon mineralization. Ecol Evol 3, 5177–5188 (2013). 24455147 10.1002/ece3.895 PMC3892327

[b36] Mo J. M. *et al.* Response of soil respiration to simulated N deposition in a disturbed and a rehabilitated tropical forest in southern China. Plant Soil 296, 125–135 (2007).

[b37] Drenovsky R. E., Steenwerth K. L., Jackson L. E. & Scow K. M. Land use and climatic factors structure regional patterns in soil microbial communities. Global Ecol Biogeogr 19, 27–39 (2010). 10.1111/j.1466-8238.2009.00486.x PMC3891896 24443643

[b38] Zhou W. P., Hui D. F. & Shen W. J. Effects of Soil Moisture on the Temperature Sensitivity of Soil Heterotrophic Respiration: A Laboratory Incubation Study. PLOS ONE 9, 10.1371/journal.pone.0092531 (2014). PMC3960259 24647610

[b39] van Diepen L. T. A., Lilleskov E. A., Pregitzer K. S. & Miller R. M. Simulated Nitrogen Deposition Causes a Decline of Intra- and Extraradical Abundance of Arbuscular Mycorrhizal Fungi and Changes in Microbial Community Structure in Northern Hardwood Forests. Ecosystems 13, 683–695 (2010).

[b40] Liu X. J. *et al.* Nitrogen deposition and its ecological impact in China: An overview. Environ Pollut 159, 2251–2264 (2011). 20828899 10.1016/j.envpol.2010.08.002

[b41] Chen H. *et al.* Effects of nitrogen deposition on carbon cycle in terrestrial ecosystems of China: A meta-analysis. Environ Pollut 206, 352–360 (2015). 26232918 10.1016/j.envpol.2015.07.033

[b42] Zhang N. *et al.* Impacts of urea N addition on soil microbial community in a semi-arid temperate steppe in northern China. Plant Soil 311, 19–28 (2008).

[b43] Riggs C. E., Hobbie S. E., Bach E. M., Hofmockel K. S. & Kazanski C. E. Nitrogen addition changes grassland soil organic matter decomposition. Biogeochemistry 125, 203–219 (2015).

[b44] Zeng D. H. *et al.* Effects of nitrogen addition on vegetation and ecosystem carbon in a semi-arid grassland. Biochemistry 98, 185–193 (2010).

[b45] Skinner R. H. Nitrogen fertilization effects on pasture photosynthesis, respiration, and ecosystem carbon content. Agr Ecosyst Environ 172, 35–41 (2013).

[b46] Lu M. *et al.* Minor stimulation of soil carbon storage by nitrogen addition: A meta-analysis. Agr Ecosyst Environ 140, 234–244 (2011).

[b47] Chen D., Li J., Lan Z., Hu S. & Bai Y. Soil acidification exerts a greater control on soil respiration than soil nitrogen availability in grasslands subjected to long-term nitrogen enrichment. Funct Ecol 10.1111/1365-2435.12525 (2015).

[b48] Chen D. *et al.* Biotic community shifts explain the contrasting responses of microbial and root respiration to experimental soil acidification. Soil Biol Biochem 90, 139–147 (2015).

[b49] Wang R. Z. *et al.* Coupled response of soil carbon and nitrogen pools and enzyme activities to nitrogen and water addition in a semi-arid grassland of Inner Mongolia. Plant Soil 381, 323–336 (2014).

[b50] Geisseler D., Horwath W. R. & Doane T. A. Significance of organic nitrogen uptake from plant residues by soil microorganisms as affected by carbon and nitrogen availability. Soil Biol Biochem 41, 1281–1288 (2009).

[b51] Wei C. *et al.* Nitrogen deposition weakens plant-microbe interactions in grassland ecosystems. Global Change Biol 19, 3688–3697 (2013). 10.1111/gcb.12348 23925948

[b52] Bradford M. A., Keiser A. D., Davies C. A., Mersmann C. A. & Strickland M. S. Empirical evidence that soil carbon formation from plant inputs is positively related to microbial growth. Biogeochemistry 113, 271–281 (2013).

[b53] Blagodatskaya E. *et al.* Microbial interactions affect sources of priming induced by cellulose. Soil Biol Biochem 74, 39–49 (2014).

[b54] Chen D. M. *et al.* Subtropical plantations are large carbon sinks: Evidence from two monoculture plantations in South China. Agr Forest Meterol 151, 1214–1225 (2011).

[b55] Kuzyakov Y. Sources of CO2 efflux from soil and review of partitioning methods. Soil Biol Biochem 38, 425–448 (2006).

[b56] Subke J., Inglima I. & Francesca Cotrufo M. Trends and methodological impacts in soil CO2 efflux partitioning: A metaanalytical review. Global Change Biol 12, 921–943 (2006).

[b57] Zhang F., Zhou G. S. & Wang Y. H. Pheonological calendar of *Stipa Krylovii* steppe in Inner Mongolia, China and its correlation with climatic variables. J Plant Ecol (Chinese Version) 32, 1312–1322 (2008).

[b58] Vance E. D., Brookes P. C. & Jenkinson D. S. An extraction method for measuring soil microbial biomass-C. Soil Biol Biochem 19, 703–707 (1987).

[b59] Bligh E. G. & Dyer W. J. A rapid method of total lipid extraction and purification. Canadian J Physio Biochem 37, 911–917 (1959). 10.1139/o59-099 13671378

[b60] Kieft T. L., Ringelberg D. B. & White D. C. Changes in ester-linked phospholipid fatty-acid profiles of subsurface bacteria during starvation and desiccation in a porous-medium. Appl Environ Microb 60, 3292–3299 (1994) 10.1128/aem.60.9.3292-3299.1994 PMC201801 16349382

