# Supplementary material for: Microbial properties explain temporal variation in soil respiration in a grassland subjected to nitrogen addition
Source: Sci Rep. 2015 Dec 18;5:18496. doi: 10.1038/srep18496 (PMC4683438; doi:10.1038/srep18496)

Microbial properties explain temporal variation in soil respiration in a grassland subjected to nitrogen addition

Yue Li [a, b], Yinghui Liu [a, c*], Shanmei Wu [a, c], Lei Niu [a, c], Yuqiang Tian [a, b]

[a] *State Key Laboratory of Earth Surface Processes and Resource Ecology, Beijing Normal University, Beijing 100875,China*

[b] *Academy of Disaster Reduction and Emergency Management, Beijing Normal University, Beijing 100875, China*

[c] *College of Resources Science and Technology, Beijing Normal University, Beijing 100875, China*

[*]Corresponding author:

Yinghui Liu

Email: lyh@bnu.edu.cn

Tel: 010-58804136

Manuscript type: Regular Articles

Supplementary information

Table S1 Soil microbial functional groups interpreted by PLFA biomarkers.

Interpretation	Specific fatty acid
Bacteria	i14:0, 15:0, i15:0, a15:0, i16:0, a16:0, 16:1w7c, 16:1w9c, 16:0 2OH, 16:1 2OH, 17:0, i17:0, a17:0, cy17:0, 17:1w8c, 18:0, i18:0, 18:1w5, 18:1w7c, i19:0, and cy19:0
Gram-positive bacteria	i14:0, i15:0, a15:0, i16:0, i17:0, a17:0, and i18:0
Gram-negative bacteria	a16:0, 16:0 2OH, 16:1w7c, 16:1w9c, cy17:0, 17:1w8c, 18:1w5c, 18:1w7c, and cy19:0
fungi	18:1w9c, 18:2w6,9c, 18:3w6,9,12c, and 20:1w9c
actinomycetes	10Me17:0 and 10Me18:0
arbuscular mycorrhizal fungi	16:1w5c
Monoenoic: saturated PLFAs	(16:1w5, 16:1w7, 16:1w9, 17:1w8, 18:1w5, 18:1w7, and 18:1w9): (14:0, 15:0, 16:0, 17:0, 18:0, 19:0, and 20:0)

Table S2 Equations used to adjust estimates of soil heterotrophic respiration (R_h) based on the effects of soil temperature (T) and soil moisture (M) with different levels of N addition

N level (g N m^{-2} yr^{-1})	Equation	R^2	P
0	$R_h = 1.264e^{0.047T}(7.265M^2 + 1.313M)$	0.83	<0.01
2	$R_h = 0.668e^{0.0069T}(1.442M^2 + 2.751M)$	0.83	<0.01
4	$R_h = 1.678e^{0.057T}(-2.414M^2 + 2.071M)$	0.92	<0.01
8	$R_h = 1.208e^{0.045T}(4.893M^2 + 1.622M)$	0.92	<0.01
16	$R_h = 2.174e^{0.050T}(-0.314M^2 + 1.192M)$	0.81	<0.01
32	$R_h = 1.960e^{0.029T}(4.939M^2 + 1.724M)$	0.94	<0.01

Table S3 Comparison of soil nutrient contents under different N addition levels in the pre-growing season (April) and the mid-growing season (July). Data shown are mean values with standard error in parenthesis. TOC is total organic carbon, TN is total nitrogen, TP is total phosphorus, C: N is total organic carbon: total nitrogen ratio. Different letters indicate significant ($P < 0.05$) differences among N levels.

Month	N level ($g\ N\ m^{-2}\ yr^{-1}$)	TOC ($g\ kg^{-1}$)	TN ($g\ kg^{-1}$)	TP ($g\ kg^{-1}$)	C: N
April	0	23.55 (2.23)	3.03 (0.09)ab	0.37 (0.03)a	7.79 (0.84)a
	2	25.97 (0.87)	2.97 (0.12)ab	0.38 (0.02)a	8.80 (0.60)a
	4	26.15 (5.05)	3.13 (0.09)a	0.40 (0.02)a	8.27 (1.36)a
	8	26.21 (2.80)	2.97 (0.12)a	0.35 (0.02)ab	8.79 (0.57)a
	16	25.48 (2.67)	3.17 (0.24)ab	0.33 (0.02)ab	8.05 (0.58)a
	32	26.71 (1.54)	3.00 (0.38)b	0.27 (0.04)b	9.29 (1.63)b
July	0	27.23 (1.01)ab	3.40 (0.15)a	0. 39 (0.02)a	8.04 (0.44)a
	2	23.88 (1.07)ac	2.93 (0.15)a	0.36 (0.01)ab	8.16 (0.31)ab
	4	28.78 (0.57)b	2.87 (0.24)b	0.40 (0.01)a	10.22 (0.38)b
	8	24.80 (2.38)abc	2.87 (0.03)b	0.35 (0.04)ab	8.63 (0.74)ab
	16	27.14 (1.70)ab	3.13 (0.09)ab	0.38 (0.02)a	8.68 (0.63)ab
	32	21.65 (0.82)c	3.07 (0.22)ab	0.31 (0.03)b	7.13 (0.57)a

Figure S1 Design of the N addition experiment. Each of six levels of N addition (0, 2, 4, 8, 16, and 32 g N m^{-2} yr^{-1}) was represented by three replicate plots (15 m × 10 m). Although the site contained 24 plots, only 18 were used in the experiment (plots without numbers in the figure were not used). Each plot had a subplot in which the heterotrophic respiration collar was located, but these subplots are not shown in the figure. At each period, plots were divided into two groups according to N level: 0, 4, and 8 g N m^{-2} yr^{-1}, and 2, 16, and 32 g N m^{-2} yr^{-1}. Three plots near each other in one group were observed on one day. Nine plots (3 plots × 3 replicates) in the same group were measured over three consecutive days.

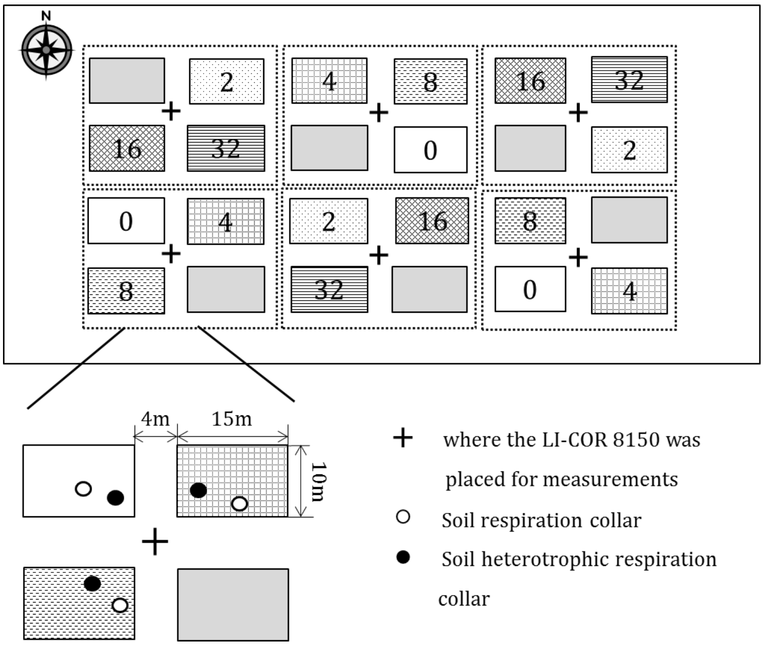

Figure S2 Soil inorganic nitrogen content in the early (a), middle (b) and late (c) growing, respectively. Different letters indicate significant ($P < 0.05$) differences among N addition levels. Errors bars represent standard error (n=3 plot replicates)

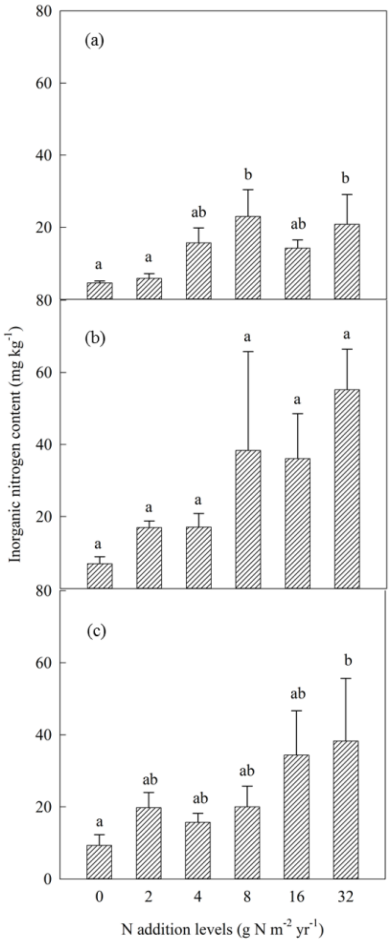

Figure S3 Soil pH value in the early (a), middle (b) and late (c) growing season, respectively. Different letters indicate significant ($P < 0.05$) differences among N addition levels. Errors bars represent standard error (n=3 plot replicates)

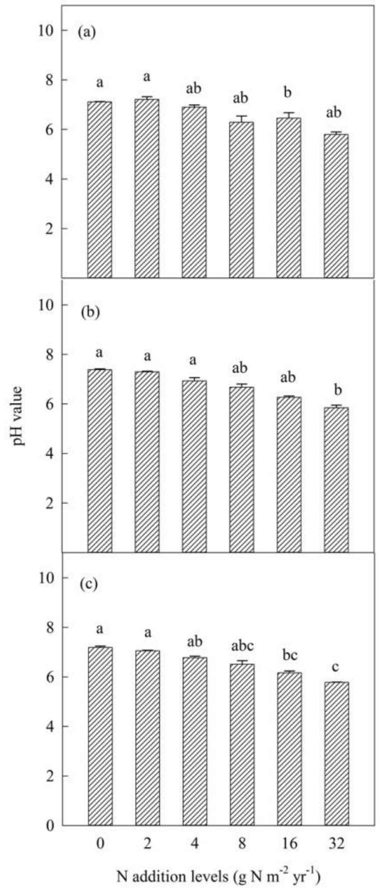

Supplement: Supplementary Information [file srep18496-s1.pdf]
